# Mass Spectrometry Imaging as a New Method: To Reveal the Pathogenesis and the Mechanism of Traditional Medicine in Cerebral Ischemia

**DOI:** 10.3389/fphar.2022.887050

**Published:** 2022-06-03

**Authors:** Yan Liang, Qiaoqiao Feng, Zhang Wang

**Affiliations:** ^1^ College of Pharmacy, Chengdu University of Traditional Chinese Medicine, Chengdu, China; ^2^ State Key Laboratory of Southwestern Chinese Medicine Resources, Chengdu University of Traditional Chinese Medicine, Chengdu, China; ^3^ College of Ethnomedicine, Chengdu University of Traditional Chinese Medicine, Chengdu, China

**Keywords:** mass spectrometry imaging, cerebral ischemia, metabolomics, spatial distribution of drugs, traditional medicines, mechanism

## Abstract

Mass spectrometry imaging (MSI) can describe the spatial distribution of molecules in various complex biological samples, such as metabolites, lipids, peptides and proteins in a comprehensive way, and can provide highly relevant supplementary information when combined with other molecular imaging techniques and chromatography techniques, so it has been used more and more widely in biomedical research. The application of mass spectrometry imaging in neuroscience is developing. It is very advantageous and necessary to use MSI to study various pathophysiological processes involved in brain injury and functional recovery during cerebral ischemia. Therefore, this paper introduces the techniques of mass spectrometry, including the principle of mass spectrometry, the acquisition and preparation of imaging samples, the commonly used ionization techniques, and the optimization of the current applied methodology. Furthermore, the research on the mechanism of cerebral ischemia by mass spectrometry was reviewed, such as phosphatidylcholine involved, dopamine, spatial distribution and level changes of physiological substances such as ATP in the Krebs cycle; The characteristics of mass spectrometry imaging as one of the methods of metabolomics in screening biomarkers related to cerebral ischemia were analyzed the advantages of MSI in revealing drug distribution and the mechanism of traditional drugs were summarized, and the existing problems of MSI were also analyzed and relevant suggestions were put forward.

## Introduction

Mass Spectrometry Imaging (MSI) is an imaging method based on mass spectrometry, which uses mass spectrometry to scan biological samples directly, not only can analyze the spatial distribution characteristics of hundreds of molecules simultaneously on the same tissue section, but also can obtain the overall distribution information of drugs and metabolites at the animal level by high-resolution mass spectrometry (Luo et al., 2017). Mass spectrometry imaging can be divided into: Matrix-Assisted Laser Desorption/Ionization Mass Spectrum Image (MALDI-MSI), Desorption Electrospray Ionization Mass Spectrum Image (DESI-MSI) and so on. Among them, MALDI imaging is the most important, but DESI imaging has more advantages in real-time clinicopathological diagnosis because of its convenient workflow (Lanekoff, Stevens et al., 2014).

As one of the young mass spectrometry techniques, MSI has a great value in many fields such as medical research, biological research, drug research and so on. It has become a hot spot in mass spectrometry research. In order to obtain high-quality mass spectrometry imaging results, from the ion source to the mass analyzer, and then to data acquisition and processing, every step needs to be done to the extreme to achieve the overall performance optimization, so as to obtain the most satisfactory results. The key issues to be paid attention to MSI are imaging rate, reproducibility and wide spectrum capability. Compared with the main clinical optical imaging such as fluorescence immune-labeling imaging, mass spectrometry imaging has many advantages, such as multiple molecular information in tissue sections, no staining and labeling, and low imaging cost. Mass spectrometry is a mass spectrometer with the highest requirements for vacuum. Therefore, the current improvement in imaging rates makes the image more accurate of biological tissues. Prior applications of mass spectrometry imaging focused on qualitative testing; such as microbial identification, nucleic acid typing, in practical application, only qualitative analysis is far from enough, the mainstream needs still to solve quantitative problems. To complete quantitatively analysis requires imaging equipment have a good repeatability, isotope labeling method can be used to assist in the realization of molecular quantitative problems. With the continuous improvement of new instrument technology, it can achieve high sensitivity in a wide mass number range, so as to ensure that the test can be completed in a wide mass number range and the information of slices can be better expressed. Both small and large biological molecules can be accurately imaged to achieve real imaging freedom.

Mass spectrometry imaging develops rapidly and can be applied to food safety, geography, environmental science, and it has great potential especially in life science and medicine. Existing imaging technologies include optical imaging, chemical imaging and so on. These techniques can only monitor the expression of one molecule and require a molecular probe. Molecules with similar structures are prone to false positive results. Molecular mass spectrometry imaging of biological tissue is helpful to solve these problems. Through the combination of mass spectrometry ion scanning technology and professional image processing software, the biological tissue section is directly analyzed, relative abundance and spatial distribution of compounds in the tissue are analyzed and studied. Mass spectrometry has been widely used for the analysis of various biomolecules in tissue sections, such as proteins, peptides, lipids and drug metabolites. In this way, the spatial distribution of potential biomarkers can be obtained, and combined with other molecular imaging techniques, it can be used for histopathological characteristics, disease diagnosis and biomarker discovery. MSI technology has found biomarkers in breast cancer, thyroid cancer, colon cancer and other cancers, becoming a powerful tool for tumors (Yun et al., 2020), normal cells and tumor cells have different cell phenotype, and different tumor cells have different membrane lipid phenotype, and the micro-environment also affects the membrane lipid phenotype of tumor cells. Studies on *in situ* detection of tumor cell membrane lipids showed that monounsaturated fatty acids, three glyceryl phosphatide and nucleotide were upregulated, inosine and a phosphatidylethanolamine were downregulated in tumor regions of gastric cancer tumor tissues (Dan et al., 2018). Neurotransmitters such as dopamine, 5-hydroxytryptamine, 3-methoxytyramine and tyrosine were efficiently detected by mass spectrometry in a derivative form (Xi et al., 2019) Mass spectrometry (MS), based on a new method of imaging drug metabolomics, provides a novel and intuitive means for the analysis of the mechanism of action or toxicological mechanism of drugs or new drugs, even new drug candidates with multiple targets or unclear targets. Using MALDI-MSI, it was found that (s)-Oxiracetam could cause changes of 15 metabolic small molecules related to glucose aerobic oxidation and other pathways (Wan, Huihui et al., 2017). In conclusion, mass spectrometry imaging has shown its advantages in tumor diagnosis, brain neuroscience, drug development, and biomarker screening and identification.

Ischemic stroke accounts for 69.6%–70.8% of cerebral stroke in China, and is the first leading cause of death and disability in China (Wu, Wu et al., 2019). Cerebral ischemia reperfusion injury refers to the phenomenon that cerebral cell injury caused by cerebral ischemia is further aggravated after blood reperfusion is restored, and the obvious changes are neuronal injury and cerebral edema (Guang, 2018). The pathogenesis of cerebral ischemia injury includes oxygen free radical damage, calcium ion overload, mitochondrial damage, neurotoxic effects, inflammatory damage and blood-brain barrier damage, they cause and effect each other and interact into a network, eventually causing nerve cell edema, injury, necrosis and apoptosis. Although intravenous thrombolysis, antiplatelet aggregation and anticoagulation therapy (Stoll and Nieswandt 2019) can be used in the treatment of stroke, reperfusion after thrombolysis can cause complications such as edema of bleeding transformed brain cells and exacerbation of brain cell dysfunction. How to alleviate cerebral ischemia reperfusion injury is one of the keys to improve the efficacy of ischemic stroke. Blood-brain barrier (BBB) plays an important role as the structural basis of cerebral ischemia. In addition, mitochondria are an important medium of cell signal and energy homeostasis, so mitochondrial dysfunction is one of the important pathogeneses of cerebral ischemia reperfusion injury (Carinci, Vezzani et al., 2021), specifically manifested as mitochondrial membrane structure was damaged and mitochondrial DNA was damaged, mitochondrial permeability transition pore (MPTP) opening up and so on, and then participate in the process of neuronal apoptosis (Wei et al., 2019). Mass spectrometry imaging has great potential in studying the spatial distribution of biomolecules in complete tissue samples.

At present, MSI has been widely used in protein recognition, biomarker discovery, medical diagnosis and other research (Chughtai and Heeren 2010). MALDI and DESI have broad application prospects in brain neuroscience research and clinical diagnosis (Xi et al., 2019). It can be used in neuroscience for the imaging of neurotransmitters and low molecular weight metabolites, lipids and peptides. The further insights into the underlying mechanisms that are going on in the nervous system, may be provided by using IMS to study the spatio-temporal dynamic distribution patterns of molecular species that coordinate neuronal function, including development, learning and memory, and cognition and behavior (Hanrieder, Phan et al., 2013). By analyzing the related physiological substances (such as lipids, dopamine and ATP in the tricarxylic acid cycle) involved in the pathogenesis of cerebral ischemia, metabolomics, distribution and mechanism of drugs in cerebral ischemia, the spatial distribution and level changes of drugs were clarified. It will play an important role in the discovery, clinical diagnosis, treatment and prognosis of cerebral ischemia, and make mass spectrometry imaging more mature in the detection of the mechanism of cerebral ischemia.

## Mass Spectrometry Imaging

Morphological abnormalities in tissue samples observed with conventional microscopy do not provide researchers with much biochemical information, and conventional biochemical techniques often lose information about tissue location. By successfully combining these two aspects of information to display objects and simultaneously determine the biochemistry and location of unknown molecules, mass spectrometry imaging can be useful in elucidating pathogenesis or identifying therapeutic targets.

### The Principle of Mass Spectrometry Imaging

Firstly, the samples tested are obtained and prepared in an appropriate way. According to the preset acquisition program, the mass spectrometer uses laser or high-energy ion beam to scan the samples, so that the molecules or ions on the surface are desorbed and ionized. Then the mass charge ratio and ion strength of each pixel ion on the sample surface are obtained by mass analyzer. The mass spectrum peak of any ion with specified mass charge ratio was searched in the mass spectrum data of each pixel with the help of mass spectral imaging software, and the two-dimensional distribution map of the corresponding molecule or ion on the sample surface was drawn by combining the signal strength of the corresponding ion and its position on the sample surface. Then, the above software is used for further data processing of the two-dimensional distribution map of continuous slices of samples to obtain the three-dimensional spatial distribution of the objects to be measured in the samples.

The key performance indexes of mass spectrometry are sensitivity and imaging rate. The key steps are tissue sectioning and matrix spraying. In order to improve the integrity of mass spectrometry imaging results, it is necessary to exert the maximum value in each of these processes. Sensitivity means the minimum number of ions the mass spectrometer can find. Factors affecting sensitivity include ionization efficiency, ion transport efficiency, and substrate (impurity) interference. Ionization efficiency refers to the degree to which a sample is ionized. Sample molecules can be easily detected by an ion detector when they are ionized enough. But different ion sources target different ranges. For example, MALDI can achieve very high ionization efficiency for both large and small molecules when the matrix is suitable, which is the reason why MALDI has high sensitivity. In contrast, DESI and SIMS plasma sources have obvious advantages in small molecule ionization, while in large molecule, the ionization ability is worse than MALDI. As the internal source (in vacuum), the ions produced by MALDI are generated in the vacuum environment of the mass spectrometer, so the ion transfer efficiency is higher. While the ions generated by external ion sources, such as AP-MALDI and DESI, in the atmosphere need to be transferred into the vacuum environment, and only less than 1% of the ions are usually transferred to the mass analyzer. It has great influence on the sensitivity of mass spectrometry, which can only be compensated by the ability of the mass analyzer. In mass spectrometric imaging, matrix interference also disturbs the mass spectrometer itself. Therefore, we can use common techniques such as chromatography-mass spectrometry, which is to remove the matrix in the sample first, and then separate the relatively pure target. It can effectively eliminate the defects of mass spectral imaging itself.

As for the scanning rate of mass spectral imaging, it generally depends on three factors: laser frequency, two-dimensional mobile platform and data acquisition. The preparation of tissue sections is mainly related to the authenticity and accuracy of mass spectrometry imaging results. Sample preprocessing is the basis of mass spectrometry. In order to reduce the time of sample preprocessing and improve the resolution and reproducibility of MALDI mass spectrometry, many researchers focus on the study of matrix free mass spectrometry. A matrix free MALDI-MS detection method was established by selective enrichment of low abundance biological small molecules and proteins through nanotechnology. A new type of ionization-assisted substrate for porous nano materials has been developed. The ionization-assisted substrate can significantly reduce the sample preprocessing time for mass spectrometry analysis, and the operation is simple.

### Acquisition and Preparation of Mass Spectrometry Image Samples

The process of sample preparation is a key link affecting the authenticity and accuracy of mass spectrometry imaging results, and its processing methods and techniques are closely related to the properties of the object to be measured and the type and state of the sample. Generally, MSI used in pharmaceutical research is mostly used to analyze animals, tissues, cells and solid preparations. Proper and rapid sample collection and fixation are the guarantee to maintain the true spatial distribution and abundance of molecules or ions in the sample. To avoid displacement and degradation of the tissue, the sample should be fixed quickly collection. The most common fixation method for tissue samples such as organs and whole animal samples is rapid freezing.

Tissue, whole animals, and solid preparations are usually sectioned. Embedding is often required for fragile tissues such as eye tissue, large samples such as whole animals and solid preparations that are not easily sectioned. The samples were transferred to the mass spectrum target by melting mounting method and adhesive tape method, respectively, by directly sticking the frozen tissue sections to the target at room temperature, or by fixing the samples on the mass spectrum target with conductive double-sided adhesive tape. Immediately after the sample is transferred to the mass target, the mass target containing the sample should be dried to keep the sample stable. The commonly used drying methods are freeze drying, vacuum drying, solvent dehydration drying and nitrogen drying. Generally, the dried samples can be directly analyzed by mass spectrometry. In the detection of specific substances to be measured in complex samples, solvent cleaning, surface enzymatic hydrolysis and chemical derivatization are often used to properly treat the analytical surface. Cleaning the mass spectrometric target with appropriate solvent can not only dehydrate and fix, but also improve the results of mass spectrometric determination.

### Mass Spectrometry Imaging Ionization Technique

The ionization methods commonly used in MSI analysis are: matrix-assisted laser desorption ionization (MALDI), desorption electrospray ionization (DESI) and secondary ion mass spectrometry (SIMS). Some ionization techniques such as atmospheric pressure infrared (APIR) mass spectrometry, nanostructure-initiator mass spectrometry (NIMS), attributes that fit MSI analysis objects are also used in MSI analysis.

MALDI-MSI first needs to uniformly deposit the organic small molecule matrix absorbing ultraviolet light on the tissue sections to be analyzed. The commonly used methods include sublimation electrospray, fluid atomization, ultrasonic spray, etc. The advantages of MALDI imaging are as follows: 1) The imaging spatial resolution is high, reaching the cell level (less than 5 μm); 2) It is suitable for the analysis of different kinds of molecules (including metabolites, lipids, proteins, nucleic acids and drugs). MALDI imaging provides mapping of biomolecules in a large mass range from intact lipids to proteins. Most are applied to brain tissue analysis. The resulting images feature hundreds of uniquely positioned compound peaks that give an indication of function and may represent neurologically important peptides and proteins.

DESI mass spectrometry imaging uses the high-speed charged fog generated by electrospray to bombard the tissue sections in an inclined direction. The charged fog drops will extract and ionize the molecules in the biological sections and enter the ion transport tube of the mass spectrometry together with them. The main advantages of DESI mass spectrometry imaging are: No need for external matrix assisted ionization, the analysis process is faster; No laser, simple ion source structure, low maintenance cost; The requirement for mass spectrometers is low, a simple ion trap mass spectrometer can support DESI imaging. Compared with MALDI imaging, DESI imaging has more advantages in real-time clinicopathological diagnosis because of its convenient workflow. However, the spatial resolution accuracy of DESI imaging is low, and it is difficult to analyze the compounds with large molecular weight (such as polypeptides, proteins and nucleic acids) (Xi et al., 2019).

Some chemical components that carry large molecular fragments cannot be imprinted by conventional mass spectrometry; Professor Nicholas Winograd of Pennsylvania State University has developed a method called SIMS that allows a complete scan of a sample in three dimensions. On the other hand, SIMS can only image small molecules. Professor Winograd has refined this approach by using a new type of SIMS beam (carbon-60 magnetic sphere) that is less chemically damaging to objects than traditional SIMS beam. The energy of C60 is comparable to other ion beams, but does not reach below the surface of the sample. So, the sample is continuously stripped layer by layer, resulting in a longitudinal plane pattern, ultimately resulting a three-dimensional molecular image. This method has good spatial resolution and can obtain the cellular characteristics and analyze the distribution of macrophages and star cells. SIMS probes can reach depths of up to 100 nm, providing nanoscale resolution.

Understanding the inner components of cells is the key to understanding that healthy cells differ from diseased cells. Akos Vertes and others have come up with a combination of two techniques to analyze living cell analysis, based on the principle that biological samples can also absorb energy directly. First, they used an atmospheric pressure infrared (APIR) MALDI laser to directly activate water in the tissue, vaporizing the sample and obtaining ionized particles that were analyzed by mass spectrometry. But not all vaporized particles are charged. Most are actually uncharged and will be missed by APIR MALDI. To capture these neutral particles, Vertes et al. used a second method, LAESI (Laser Ablation Electrospray), which captures a large number of charged droplets and then re-ionizes them. By treating the entire sample and combining the two methods, more molecules can be covered and the analysis quality is higher. Unlike the usual mass spectrometry process, Verte’s method also adds height to the image to enable 3D metabolite imaging, allowing researchers to obtain a natural configuration.

Mass spectrometry has great potential in the detection of molecules, Dr. Gary Siuzdak has developed a new technique called NIMS, which analyses very small regions with great sensitivity, allowing the analysis of peptide arrays, blood, urine and individual cells, as well as tissue imaging. NIMS uses lasers or ion beams to vaporize materials from nanoscale vesicles, overcoming the deficiencies of conventional mass spectrometry methods that lack the required sensitivity and require matrix molecules to ionize the analyzed objects. Many types of small molecules, such as lipids, saccharides, and steroids, can be analyzed in this way. The comparison of the characteristics of different imaging technologies is shown in Table 1.

**TABLE 1:** Comparison of imaging techniques.

Classification	Description	Advantage	Limitation	Application
Imaging mass spectrometry	MALDI-MSI	High resolution	Sample pre-treatment is required, need to select a matrix	Lipids, metabolites, proteins, drugs
	DESI-MSI	No prior treatment, easy operation	Low resolution accuracy, it is difficult to analyze the compounds with large molecular weight	Clinical analysis, cancer research, environmental science
	SIMS	High spatial resolution and little damage to samples	Only image small molecules	Micromolecule
Optical Image	Fluorescence imaging	Specificity is strong	Need to be dyed	Histological analysis, protein
Chemical imaging	PET (Positron Emission Computed Tomography)	Whole body imaging	Need to mark	Clinical analysis: tumor, cardiovascular
	MRI (Magnetic Resonance Imaging)	Whole body imaging	Long scanning time and low spatial resolution	Clinical human imaging

### Optimization of Mass Spectrometry Imaging Methodology Related to Cerebral Ischemia

Recent advances in mass spectrometry emphasis the methodology. Freezing or formalin fixation, sample preparation of tissues, and matrix selection all contribute to the improvement of mass spectrometry imaging techniques. Therefore, some aspects of processing sample preparation, including washing and desalination, matrix selection for mass spectrometry and its deposition (Chatterji and Pich 2013) are introduced to help more accurately explore the pathogenesis and the therapeutic effect of drugs on disease. Certain substrates have shown excellent performance in the analysis of a variety of small molecular metabolites, including free fatty acids, amino acids, peptides, antioxidants, and phospholipids. N-phenyl 2-naphthylamine (PNA) has strong ultraviolet absorption capacity, low background interference and salt tolerance in small molecular range. It was found that the use of 3-Aminophthalhydrazide (Luminol) (Li, Sun et al., 2019), N-phenyl 2-naphthylamine (PNA) (Liu, Zhou et al., 2018) as a new matrix for MALDI mass spectrometry can better detect endogenous metabolites in mouse brain. The abnormal metabolism in MCAO mice can reveal the changes of 105 metabolites in the ipsilateral hemisphere. The metabolites in the brain of normal mice were detected by mass spectrometry, lipids and other molecular types can be accurately detected, the spatial distribution of metabolites in the mouse brain can be gotten, the tissue-specific spatial distribution of gangliosides was visualized, they were mainly distributed in HIP, CTX and striatum, with less distribution in cerebellum, revealing the spatial distribution of lipid substances. Small molecules such as fatty acids and nucleosides were also visualized, mainly distributed in the cerebellar granular layer and striatum. Many molecular species show significant changes in coronal sections of infarcted mouse brains. In anion mode, small molecules (such as AMP, ADP, GMP, GSH, and FAs) signal significantly decreased in the ischemic region, the ischemic region also altered ganglioside signaling, and after reperfusion, the highest levels of GM1, GM2, and GM3 were observed in MCAO mouse models. The changed metabolites were identified by relative quantitative and statistical analysis. The results show that the newly prepared matrix has the characteristics of low cost, strong UV absorption, high salt tolerance and less background signal. In the low-quality range, the spatial distribution of various small molecular metabolites, including metal ions, amino acids, carboxylic acids, nucleotide derivatives, peptides and lipids, can be observed directly. The matrix effect of nano-spray desorption electrospray ionized MSI (NANO-DESI MSI) can also be examined by mouse brain tissue. The strength of the sodium and potassium adducts of endogenous phosphatidylcholine (PC) species was normalized at a constant rate with the strength of the corresponding adducts of the PC standard supplied by the nano solvent. Significant differences in sodium and potassium concentrations in the ischemic region compared with healthy tissue distinguish the two matrix effects. Two types of matrix effects can be compensated by normalizing the signal corresponding to the internal PC into a standard signal. This method can effectively compensate for signal changes (Lanekoff, Stevens et al., 2014).

Due to the diversity of lipids, the analysis and imaging of multiple lipids by MALDI-MSI is a huge challenge. Ten lipids from the brain were analyzed when polyvinylpyrrolidone (PVP) covered silver nanoparticles (AgNPs) were first used as a matrix for MALDI-MSI. Analysis included fatty acids and their derivatives, sterols, CPA, LPA, and PA, LPE, and PE, LPC, and PC, PS, Cers, SM, and MAG and DAG, and other small metabolites. Due to the presence of large amounts of silver ions on the surface of PVP capped AgNP, compounds with poor ionization efficiency, such as FA and sterols, can be detected. MALDI-MSI analysis of mouse brain based on PVP AgNPs showed that lipid distribution in mouse brain substructure was related to its biological function. It was also found that K + adducts of most unsaturated fatty acids, prostaglandins, CPA, vitamin A, neuraminidase, 5-OH-tryptophan and most phospholipids (PA, LPE, PE, PC, PS) and SM were significantly downregulated in the ischemic zone and saturated fatty acids. The Na + adducts of Cers, hexyl carnitine, stearaldehyde, phospholipids (LPA, PAs, LPE, PEs, LPC, PCs) and SMs were highly expressed in the damaged site. These novel findings may be important to elucidate the mechanisms of disease. Using PVP capped AgNP as a matrix for MALDI MSI could be a powerful tool in histopathological and pathological studies (Guan, Zhang et al., 2018). The SERS spectroscopy which is sensitive to the state of molecular vibration can provide the molecular visualization without labels, and the self-assembled nanostructure of boehmite can be used to produce the homogeneous SERS active substrate on a large area. The matrix, known as “gold-nano-coral” (GNC), is based on its coral-like shape. The transparency of the boehmite allows measurements to be made from the back of the substrate as effectively as from the front. Tissue imaging was performed using ischemic mouse brains glued to the GNC substrate. By constructing a intensity map using differential bands from two metabolically distinct regions, namely the ischemic core and the contralateral control region, the findings were as determined by imaging mass spectrometry: the adenine ring vibratory band clearly delineates ischemia, with degradation of high energy adenine phosphate nucleotides in the core. This detection function makes SERS technology particularly promising for revealing acute energy disorders in tissues (Yamazoe, Naya et al., 2014). New substrates have great potential applications in biomedical research.

Tissue preparation is the key to successful MALDI-MSI experiments. Rapid tissue change in animal models presents significant challenges for the analysis of peptides and metabolites in MSI. The effect of the thawing duration of tissue sections on the stability of metabolites was determined by a modified method of tissue fixation with thermal stability *in vitro* and freezing *in situ* in a mouse model of middle cerebral artery occlusion (MCAO) after stroke. Higher stability and biomolecular visualization was demonstrated when using frozen mice compared to thermal stabilization. The results were further improved when funnel freezing was combined with rapid thawing of brain slices (Mulder, Esteve et al., 2016). The thin layers near the rat brain were imaged by Fourier transform infrared (FT-IR) spectroscopy and laser ablation inductively coupled plasma mass spectrometry (LA-ICP-MS). Each hyperspectral data set was fused into a multi-sensor hyperspectral data cube and multivariate analysis was performed. Using models based on PLS-DA or RDF algorithms to identify and classify target regions (IZ, PIZ, WM, and GM), multi-sensor hyperspectral imaging can deepen the understanding of ischemic brain biochemical processes and automatically identify different types of biochemical process organizations (Balbekova, Lohninger et al., 2018).

## The Physiological Substances Related to the Pathogenesis of Cerebral Ischemia Were Detected by Mass Spectrometry

The pathophysiological processes of cerebral ischemia are complex and multimodal, and understanding the underlying pathologic processes can help guide future research and improve the prognosis of some patients. Compared with other tissues, the brain is affected by many factors, such as strict dependence on energy substrates such as glucose and oxygen, automatic regulation of blood flow, blood-brain barrier, etc. making cerebral ischemia a very heterogeneous and multifactorial process. Lipids, especially phospholipids, are fundamental to the structure and function of the central nervous system. Monoamine neurotransmitters are released by specialized neurons that regulate behavior, movement, and cognitive function. The distribution and dynamics of monoamine may be related to the mechanism of cerebral ischemia, and energy-related metabolites have been recognized as useful markers of energy crisis during focal ischemia.

### Lipids

Mass spectrometry imaging can be used to detect the cerebral lipid dynamics of cerebral ischemia-reperfusion models, suggesting potential biomarkers and pharmacological targets. Phospholipids are highly susceptible to free radical attack and oxidative modification and play an important role in brain function and neurodegenerative diseases. Phospholipids are the main components of biofilms, which are divided into glycerophospholipids and sphingomyelins, which are composed of glycerol and sphingomyelins respectively. The dynamic changes of phospholipid levels after cerebral ischemia can guide the discovery of new biomarkers and therapeutic targets to reduce the degree of infarction in the brain after ischemic stroke. The application of phospholipid imaging has been demonstrated based on the changes in lipid distribution and levels in the early stage of cerebral infarction, which can help to increase the understanding of the degree of infarction.

MALDI-IMS analysis and imaging studied ischemic mediated lipid changes in rats, which greatly simplified the spectral representation of tissue lipids. There have been some imaging studies on the spatial distribution and level changes of phospholipids in ischemic brain using MALDI-IMS. MALDI-TOF-MS/MS was used to characterize the different types of phospholipids in the brain of rats in the stroke model, and these phospholipids showed a unique distribution between the normal and damaged areas of the brain. Research shows ischemia/reperfusion injury in rats changed especially the CA1 domain in hippocampus of phospholipids appearance, confirm the hemolysis phosphatidyl choline, phosphatidyl choline, phosphatidyl ethanolamine and sphingomyelin changed. Compared with normal brain regions, PC was decreased in focal ischemic regions. In addition to a sustained decrease in phosphatidylcholine (PC) levels, PC images of all saturated, co-saturated, and monounsaturated fatty acids (MUFA) residues showed parenchymal increases after ischemia. Ischemia reduced the abundance of PC 16:0/20:4 and PC 16:0/22:6 and disrupted the stratification of the former. However, images of phospholipid in the brain parenchyma showed a continuous decrease in sphinomyelin levels after ischemia, parallel to the increase in ceramide levels (Wang, Wu et al., 2012). Lysophosphatidylcholine (LPC) was significantly increased in focal cerebral ischemia. In IMS analysis, PC and LPC changes can be seen outside the damage area. PCs is hydrolyzed by phospholipase A2 (PLA2) to produce LPCs. LPC is produced during injury after cerebral ischemia and activated by PLA2, while PC is one of its precursor molecules (Koizumi, Yamamoto et al., 2010). The ion abundance of ceramides, a lipid associated with apoptosis, was greatly increased in brain regions. MALDI-IMS detected a significant increase in ceramides in the damaged area, a significant loss of potassium adduct signal in the most abundant phosphocholine molecular species (PC), and a corresponding increase in sodium adduct ions. This change in PC alkali attachment ions is thought to be the result of edema and extracellular fluid inflow due to loss of Na/K ATPase caused by injury (Hankin, Farias et al., 2011). IMS detected significant molecular changes in the same hippocampal CA1 domain after cerebral ischemia: Hyperacute PC increased, subacute to chronic PC increased. Combined histopathology showed neuronal death associated with CA1 gliosis, inflammation, and the accumulation of activated microglia. It is shown that IMS can provide comprehensive and supplementary information on the mechanism of hippocampal CA1 cell death after global cerebral ischemia (Miyawaki, Imai et al., 2016). Other studies have shown that low ATP production and uncontrolled ion flow across the membrane trigger the production of different types of phospholipases, which in turn produce excess LPC, DAG, and other second messengers during ischemia. The changes in the composition of total phospholipids caused by brain ischemia can be analyzed by IMS data model to understand the directly related damaged tissues. The results showed that the identified phospholipids could be used as a phospholipid biomarker for ischemic injury (Shanta, Choi et al., 2012). These studies reveal the value of MALDI IMS in detecting biochemical changes in tissue lipids and will provide the data needed to ultimately understand the biochemical mechanisms associated with tissue damage.

MALDI-IMS can also detect gangliosides which are membranous lipids enriched in the central nervous system. A full understanding of how gangliosides regulated in the central nervous system is critical because of the importance of the aging brain and several neurodegenerative injuries and diseases, which refer to a chronic progressive disease resulting from the degeneration and loss of neurons in the brain and spinal cord such as stroke, Alzheimer’s disease, Parkinson’s disease, Huntington’s disease, and several lysosomal retention disorders, including Tay In Sach’s disease (Wang and Whitehead 2020), gangliosides play an important role. Mass spectrometry can distinguish gangliosides not only by their oligosaccharide composition, but also by their carbon length within the sphingosine base. After MCAO reperfusion injury, the expression ratio of various gangliosides detected by MALDI-MSI was significantly different between the ipsilateral and contralateral cortex. MCAO resulted in transient induction of GM2 and GM3 signals in the ipsilateral hemisphere at the boundary of infarcted tissue (Whitehead, Chan et al., 2011). In addition, the expression profiles of series A gangliosides GD1a, GM1, GM2, and GM3 have been found to change in the presence of ischemic injury. A significant increase in GM2 was observed in ischemic regions of stroke rats (Caughlin, Hepburn et al., 2015). In conclusion, changes in ganglioside levels reveal GM2 and GM3 as physiological markers involved in the pathogenesis of stroke.

Using the SIMS-MSI technique to identify lipids at 4, 8, and 24 h after ischemic stroke in mice with middle cerebral artery occlusion, the associated lipid content varied between different brain regions. According to the study, the infarct core, penumbra (tissue that has not yet converted to core) and surrounding healthy tissue vary in lipid content 24 h after tMCAO. Phosphatidylinositol 4-phosphate is seen in the penumbra. 2D renderings of multiple phosphatidylcholine (PC) and Lyso-PC isotypes show infarct evolution. High-resolution secondary ion mass spectrometry, used to assess the sodium/potassium ratio, showed a significant increase in sodium content and a significant decrease in potassium species in the ischemic region (core and penumbra) at 24 h after tMCAO, compared with healthy tissue. In transgenic mouse models with increased susceptibility to ischemic stroke, more significant differences in sodium/potassium ratios were found between penumbra and healthy regions (Mulder, Ogrinc Potočnik et al., 2019).

DESI-MSI can not only analyze the temporal and spatial changes of liposomes in the brain tissues of chronic cerebral hypopfusion rats with bilateral common carotid artery occlusion (BCCAO) during the progression and regression of focal cerebral inflammation, but also detect the differences between ischemic and non-ischemic brain lipids, and also detect the involvement mechanism of ion receptors. Studies have shown that the levels of arachidonic acid (ARA), docosahexaenoic acid (DHA), dihomo-C-linolenic acid, hydroxyl eicosenotrienoic acid (HETE) -ALA and glyceryl phosphatide ethanolamine in the hippocampus and cortex of model animals were significantly reduced compared with control animals. Capric acid increased after 30 days in the model group. C-linolenic acid ion and stearic acid have the highest recognition potential. These findings suggest that lipid dynamics are altered in BCCAO-induced chronic ischemia in rats and indicate potential biomarkers and pharmacological targets (Severiano, Oliveira-Lima et al., 2020). N-methyl-d-aspartic acid (NMDA) receptors are also involved in processes associated with neurodegenerative diseases and brain trauma, as key mediators of pathologic glutamate-driven excitatory toxicity and subsequent neuronal death in acute ischemic stroke (Dingledine, Borges et al., 1999). These receptors were analyzed in mouse brain slices *via* DESI-IMS. Studies on the spatial synthesis of N-acylphosphatidylethanolamine (NAPE) and N-acylphosphatidylethanolamine (NAE) during ischemia-reperfusion and their spatial degradation compared with other phospholipids showed that the content of many NAPE species increased significantly throughout the infarct area, and the content of NAE increased slightly. In the ischemic region, sodium adducts for phosphatidylcholine and lysophosphatidylcholine accumulated, while potassium adducts for phosphatidylcholine disappeared, indicating Na/K pump damage. Free fatty acids, such as arachidonic acid and docosahexaenoic acid, are more abundant in the periphery than in the center of the ischemic region and exhibit a different spatial distribution. NAPEs are synthesized throughout the ischemic region, and free fatty acids can be found in the periphery of the ischemic region (Janfelt, Wellner et al., 2012). In rats with focal cerebral ischemia, n-acyl-phosphatidylethanolamine, lysophosphatidylcholine and ceramide accumulated within 24 h, while sphingomyelin disappeared. At the later stage of decomposition, there are bis(monoacylglycero)phosphate (BMP), 2-aracidacylglycerol, ceramide phosphate, sphingosine-1-phosphate, lysophosphatidylserine and cholesterol esters. At day five to seven, dihydroxyl derivatives of docosahexene and docosaentaenoic acid, some of which may be decomposing agents, such as hemolysin, and BMP co-located with macrophage biomarker CD11b and cholesterol esters were found in the damaged area. These results suggest that BMP and N-acylphosphatidylethanolamine may serve as biomarkers for phagocytosis of macrophages/microglia and dead neurons, respectively (Nielsen, Lambertsen et al., 2016).

Mass spectrometry can also be used in combination with other techniques to increase the reliability of test lipids, such as magnetic resonance imaging (MRI) and positron emission tomography (PET). MRI was used to image the rat brain after ischemic stroke to locate the infarct area, and PET was used to image neuroinflammation and neurodegeneration. Immediately after the PET scan, the rat brain was frozen and sliced to image with MALDI-MSI. Three months after stroke, PET imaging shows little detection of neurodegeneration and nerve inflammation, indicating that the brain has stabilized. MALDI-MSI revealed significant differences in lipid distribution (such as phosphatidylcholine and sphinomyelin between scar and healthy brain), indicating that the recovery process is still ongoing. The data demonstrate the ability to combine MALD-MSI with *in vivo* PET to image different aspects of stroke recovery (Henderson, Hart et al., 2018), contributing to the reliability of the results. MSI can also be used to identify biomarkers of fatty acid metabolism in clinical testing. A differential model was established between ischemic stroke patients and healthy subjects by UHPLC mass spectrometry to identify biomarkers of fatty acid metabolites in ischemic stroke. This study may help to understand the role of fatty acid metabolites in stroke occurrence (Zhang, Ma et al., 2020). GluN2D^−/−^ mice were studied in ischemic stroke model using MALDI FT-ICR mass spectrometry, and the role of GluN2D subunit of NMDA receptor in cerebral ischemia was identified. Analysis of tissue sections by MALDI FT-ICR mass spectrometry revealed an increase in several calcia-related species (Vitamin D metabolites, LysoPC and several PS species) in wild-type mouse brain tissue. In addition, GluN2D^−/−^ mice also showed elevated PC and decreased DG, indicating decreased free fatty acids released by cerebral ischemia. These trends suggest that GluN2D^−/−^ mice show a higher rate of neurological recovery and neuroprotection against ischemic stroke compared to wild-type mice. The neuroprotective cause may be the result of increased PGP in knockout mice, which contributes to increased cardiolin synthesis and reduced sensitivity to apoptotic signals (Andrews, Donahue et al., 2020). A combination of analytical methods, including mass spectrometry, provides detailed information about the content and distribution of lipids and their oxidation products, and thus constitutes an emerging field of oxidized lipidomics. Lipid-specific oxidative modifications are critical for many cellular functions, disease states, and responses to oxidative stress. In particular, the selective oxidation of mitochondria-specific phospholipids, cardiolipins, is associated with the occurrence and development of apoptosis in injured neurons, suggesting a new target for drug discovery (Sparvero, Amoscato et al., 2010).

### Neurotransmitters

As a neurotransmitter, dopamine regulates many physiological functions of the central nervous system. The use of DESI-MSI in coronal and sagittal sections of rat brain allows direct spatial monitoring of neurotransmitters and choline without derivatization agents or deuterium agents. Amino acids, γ -aminobutyric acid (GABA), glutamate, aspartic acid, serine, and acetylcholine, dopamine, and choline have been successfully imaged by DESI in combination with a mixed quadrupole mass spectrometer, which can determine the spatial distribution of the analyzed compounds in different brain regions. Previously, aspartic acid and serine, which were rarely detected by mass spectrometry, can also be imaged with the current mass spectrometry technology to clarify their spatial distribution. DESI-MSI can directly reveal the spatial distribution of neurotransmitters in rat brain slices under simple environment, and can be used for spatial screening of neurotransmitters. DESI-MSI applied to neurotransmitter imaging (Fernandes, Vendramini et al., 2016) has the advantages of low environmental requirements and no need for stroma, and can generate serotonin (5-HT), dopamine (DA) and norepinephrine (NE) levels in mouse brain atlas. The resulting atlas revealed several nuclei rich in 5-HT and catecholamine (DA or NE), and identified 5-HT and NE in the paraventricular nucleus (PVT) of the thalamus. *In vivo* analysis of 5-HT fluctuations in response to acute tryptophan depletion and the injection of isotopically labeled tryptophan revealed a strong dynamic association between the rapt nucleus, PVT, and amygdala, but not other nuclei. The findings suggest the presence of a highly dynamic 5-HT-mediated PVT gap, which may play a role in the brain monoamine system. Direct mapping of brain monoamines is extremely challenging because it requires quantitative detection and spatial resolution. Mass spectroscopic imaging of processed brain monoamines in combination with other methods such as histochemical fluorescence techniques can make the analysis much easier and faster (Sugiyama, Guerrini et al., 2019). The matrix-assisted laser desorption/ionization mass spectrometry imaging in combination with multivariate analysis could detect age-related increases in 3,4-dihydroxyphenylacetaldehyde and histamine, indicating oxidative stress and aging deficits in astrocytes, it may help understand the central effects of neuroactive substances (Elva et al., 2022).

### Tricarboxylic Acid Cycle ATP-Related Physiological Substances

Energy metabolism is closely related to the pathogenesis of cerebral ischemia. MALDI-MSI can be used to detect changes in ATP metabolism during oxidative phosphorylation of cell mitochondria. During cerebral ischemia, restricted transport of oxygen and glucose affects ATP synthesis and metabolism, glycolysis, TCA cycle and related metabolic pathways. Interference with the inflow and outflow of metal ions and changes in antioxidant substances (Yousuf, Atif et al., 2011) also occur, accompanied by acidosis (Siesjö 1988), inflammation (Jin, Yang et al., 2010), excess production of reactive oxygen species (Schmidley 1990) and eventually neuron death (Unal-Cevik, Kilinç et al., 2004). Previous studies have reported changes in ATP, adenosine, hypoxanthine, inosine and PCr levels after MCAO. According to the study of Huihui Liu et al., 1, 5-naphthalenediamine (1, 5-Dan) hydrochloride was prepared and used for MALDI-MSI of small molecules in mouse liver, brain and kidney. It was found that 19 endogenous metabolites involved in the metabolic network, such as ATP metabolism, the tricarboxylic acid (TCA) cycle, glutamate-glutamine cycle and malate-aspartic acid shuttle, as well as metal ions and phospholipids, and antioxidants, changed relatively significantly 24 h after MCAO. The results were in good agreement with those obtained by MRM-MS analysis (Liu, Chen et al., 2014). The local response to energy metabolism during cerebral ischemia is too uneven to decipher the redox distribution between the hypoxic core and adjacent salvable regions such as penumbra. The temporal and spatial variations of adenosine and NADH in a mouse middle cerebral artery occlusion model can be revealed by MSI combined with quantitative metabolomics. Unlike the ATP-reduced core, the penumbra shows abnormally elevated ATP, despite limited blood supply. Notably, the elevated NADH in the ischemic region was clearly defined by the ATP-consuming nucleus. The results show that metabolism in ischemic penumbra does not respond passively to complex circulation, but actively compensates for energy expenditure (Hattori, Kajimura et al., 2010).

Neonatal hypoxic ischemic encephalopathy is a kind of cerebral ischemia. Brain imaging and blood tests were performed in neonates with hypoxic ischemic encephalopathy using nuclear magnetic resonance spectroscopy and spectroscopic analysis to assess acyl carnitine levels as a marker of neonatal cerebral ischemia prognosis. Reduced levels of long-chain acylcarnitine were found in neonatal patients with hypoxic ischemic encephalopathy (López-Suárez, Concheiro-Guisán et al., 2019). Mass spectrum imaging can also be used to study the immediate (0 h), subacute (36 h), and late (144 h) responses of neonatal rat brain to experimental HI (hypoxic-ischemic encephalopathy) injury, showing abnormal plasma membrane distribution of Na +/K + ions in areas affected by edema at the striatal level. In the subacute phase, the initial accumulation of a series of N-acylphosphatidylethanolamine (NAPE) molecules was detected in the HI brain, and types of cytotoxicity and angiogenic edema were detected. Abnormal distribution of the monostatic acid gangliosides GM2 and GM3 was observed in two-thirds of the heavily infected brain regions. These specific molecular changes may be further used in neonatology practice to propose and test new therapeutic strategies for neonatal HI encephalopathy (Luptakova, Baciak et al., 2018). In this study, hypothermia is known to improve the neurological function after perinatal cerebral hypoxia and ischemia, and the spatial changes of target metabolites in the brain are revealed by targeting metabolomics in combination with quantitative imaging mass spectrometry. Analysis of 107 metabolites showed that low temperatures reduced the carbon biomass associated with acetyl groups, such as pyruvate and acetyl COA; Instead, it increases deacetyl metabolites such as carnitine and choline. Quantitative MSI showed that hypothermia decreased acetylcholine levels in the hippocampus and amygdala. This reduction is associated with an inverse increase in carnitine in the same anatomical area. These findings suggest that hypothermia achieves its neuroprotective effect by synergistically inhibiting the acetyl-coA-mediated cellular acetylation state, which resides at the junction of glycolysis, amino acid catabolism and ketone catabolism (Takenouchi, Sugiura et al., 2015). The treatment of neonatal hypoxic ischemic encephalopathy with topiramate by MSI was studied. Of the one hundred and ten children, fifty-seven were given topiramate and fifty-three were given placebo. Pharmacokinetics of topiramate, energy-related Krebs cycle intermediates, and lipid peroxide-recognition biomarkers were evaluated using mass spectrometry and MRI to assess brain injury. Topiramate reached the therapeutic range of 37.5% and 75.5% of patients at 24 h and 48 h, respectively. Demonstrated that topiramate reduced seizures in patients who reached treatment levels in the first few hours after the initiation of treatment (Nuñez-Ramiro, Benavente-Fernández et al., 2019). Mass spectrum imaging provides a new basis for the process of drug treatment of diseases.

The series of pathological changes of hypoxia induced by cerebral ischemia has been the focus of research. Mass spectrometry imaging can be used to verify the pathogenesis and the effectiveness of treatment. Hypoxic preconditioning (HPC) has a protective effect on hypoxic injury. Matrix-assisted laser desorption ionization time-of-flight mass spectrometry (MALDI-TOF/TOF-MS) was used to identify the differential expression of key proteins in the mouse brain during HPC by Western blot. Consistent results were observed for pro-protein, ATP synthase subunit, malate dehydrogenase, guanine nucleotide binding protein subunit β-1 and proteasome subunit α2 types in western blotting. Proteins associated with ATP synthesis and the limonate cycle were downregulated, while proteins associated with glycolysis and oxygen binding were upregulated. Proteomic analysis of the mouse brain following HPC provides further insights into the molecular pathways involved in HPC’s protective effects, and these findings provide new insights into the mechanisms underlying hypoxia and HPC (Cui, Zhou et al., 2015). To investigate whether heme oxygenase two plays a protective role against stroke in mice, the mass of the ipsilateral and contralateral hemispheres of the MCAO models was analyzed by quantitative imaging mass spectrometer to optimize the distribution of local metabolites. Under deoxygenation, the blood flow velocity of precapillary arterioles in mice destroyed by heme oxygenase two was significantly increased, and the metabolic intermediates of central carbon metabolism and glutamate synthesis in the brain were increased. This suggests a higher metabolic requirement for inducing hypoxemia in these mice. In response to local ischemia, the heme oxygenase-2 damaged mice showed a larger ischemic core region, consistent with a significant reduction in contralateral hemisphere energy metabolism in the penumbra. Ultimately, these findings suggest that heme oxygenase two is not only involved in the mechanism that protects impaired ipsilateral hemisphere energy metabolism in ischemic contralateral hemisphere hyaline cleavage rate (Goto, Morikawa et al., 2018).

## Mass Spectrometry Imaging and Metabolomics

Metabolomics is a global systematic biology method. By observing the changes of metabolites or the changes of metabolites over time after stimulating or disturbing organisms, Metabolomics can identify small molecular substances produced by cell tissues and microorganisms in high throughput (Nicholson, Connelly et al., 2002; Graham, Rey et al., 2018), which is a technique for studying metabolic pathways. MSI is helpful to study the metabolic pathways occurring in drug treatment of diseases, and plays an important role in the detection of metabolites produced by various metabolic pathways and biomarkers.

MALDI-MSI revealed the metabolite changes in transient middle cerebral artery occlusion (tMCAO) mouse. Three metabolites creatine (Cr), phosphocreatine (P-CR) and ceramide (Cer) are associated with cell life and death. MSI revealed the presence of several ionic substances and revealed spatial variations of some metabolites identified as precise substances, including Cr, P-Cr, Cer, phosphatidylcholine, l-glutamine and l-histidine. After tMCAO, P-Cr and Cer accumulated in the ischemic core and surrounding regions over time after the ischemic attack. Upregulation of P-Cr and Cer was detected 1 h after tMCAO. P-cr and Cer can be used as neuroprotective therapies as biomarkers for cerebral ischemia (Abe, Niizuma et al., 2018). The results of the cerebral infarct size, cerebral edema, and behavioral tests showed that the brain damage was severe on the first day of I/R, and brain function significantly recovered from day 7, while the brain damage intensified from day 7 to day 28. In addition, MALDI-MSI analysis showed that ATP, glucose and citric acid expression were highest at day 7 during I/R, suggesting that energy metabolism and oxidative phosphorylation play an important role. In addition, the targeted metabolomics, according to the results of isocitrate levels associated with cerebral ischemia reperfusion, oxygen glutaric acid/proportion and glutamate pyruvate levels are highest at the 7th day of TCA cycle, 7 days after the prompt ischemia-reperfusion instantaneous spontaneous recovery may with the period of ischemic cerebral oxidative phosphorylation and energy metabolism in the brain. In conclusion, the results suggest that some small molecule metabolites are involved in brain injury and functional recovery during brain I/R, which has important implications for the development of therapeutic drugs and diagnostic markers (Cheng, Yang et al., 2020). Advanced targeted MS images can help carry out more accurate metabolite analysis. The MS images can reveal differences in abundances of selected target metabolites between cancerous and noncancerous regions of the kidney tissue by infrared (IR) laser ablation-remote-electrospray ionization (LARESI) platform. Combined with a tandem mass spectrometer (MS/MS) operated in selected reaction monitoring (SRM) or multiple reaction monitoring (MRM) modes can be developed and employed for imaging of target metabolites in human kidney cancer tissue (Joanna et al., 2020), and laser desorption/ionization MS imaging combined with silver nanoparticle-enhanced target enabled rapid visualization of the differences between the clear cell renal cell carcinoma and the healthy part of the kidney tissue (Adrian et al., 2018). Multiple metabolic alterations induced by normal aging in specific regions of mouse brains can be visualized by integrating Fourier-transform ion cyclotron resonance mass spectrometry imaging and combined supervised and unsupervised machine learning models. The imaging approach helped visualize heterogeneous age-induced metabolic perturbations in mitochondrial function, neurotransmission, and lipid signaling (Theodosia et al., 2021).

Using proteomics to identify changes in proteins in MCAO model, identification of proteins involved in cerebral ischemia may allow the discovery of putative biomarkers or therapeutic targets for ischemic stroke. MALDI-MS was used to study the distribution of proteins in mouse brain after ischemic injury and to identify proteins involved in brain injury. Brain slices were analyzed by MALDI-TOF and the infarct (IC) and contralateral (CL) regions were compared. The ion profiles of the related M/Z values were obtained. The protein was identified and the abundance of IC and CL was different. Thirteen of them were found to have increased infarct area. Identification and analysis confirmed that the expression of ATP5i, COX6C and UMP-CMP kinases in IC was altered compared with CL (Llombart, Trejo et al., 2017). The MALDI-MS is capable of spatially resolving the unique distribution of multiple metabolites, including nucleotides, cofactors, phosphorylated sugars, amino acids, lipids, and carboxylic acids, in normal mouse brain tissue, demonstrating its suitability for obtaining chemically diverse metabolite spectra in a single mammalian cell. Differentially expressed 33 protein spots in plasma samples after focal cerebral ischemia/reperfusion were found to belong to eight proteins. A group of acute phase proteins (A2 macroglobulin, complement C3, inter-α-trypsin inhibitor heavy chain H3, serum albumin, haptoglobin, and thyroxine transporter proteins) showed significant changes (Chen, Vendrell et al., 2011). In addition, MS imaging in a rat model of transient cerebral artery occlusion combined with metabolic pathway analysis visualized the temporal and spatial behavior of metabolites in the central metabolic pathway regulated by ischemia-reperfusion. These findings highlight the potential application of *in situ* metabolomics imaging in visualizing the temporal and spatial dynamics of the tissue metabolome, which will facilitate biological discoveries in preclinical and clinical settings (Miura, Fujimura et al., 2010).

## Mass Spectrometry Imaging Was Used to Study Drug Distribution

The integrated analysis of mass spectroscopy and high sensitivity omics makes it more convenient to study the spatial imaging of molecular tissue distribution. Cerebral ischemia affects the blood supply of the brain and causes abnormal metabolism of brain tissue, thus causing neurological function to be damaged and abnormal. So far, MSI of neurotransmitters has been performed mainly by MALDI. In this study, Moises Freitas-Andrade used MALDI-MSI to investigate the astrocyte conjunctions 43 (Cx43) gap conjunctions (GJ) associated with neuron survival. Cx43 may be a potential target for stroke therapy. They investigated the role of Danegapide (ZP1609), an antiarrhythmic dipeptide that specifically enhances GJ conductance, in two different rodent models of stroke. In the study, Danegapide increased Cx43 conjugation in astrocytes. Pretreated with MALDI-IMS, the NCE of danegapide in brain tissue sections was detected 1 h after reperfusion, indicating the successful transport of the dipeptide on the blood-brain barrier. In addition, the use of danegapide in a new mouse model of cerebral ischemia/reperfusion showed a significant reduction in infarct volume. This provides evidence for the efficacy of danegapide in the treatment of ischemic/reperfusion stroke. The mass spectrum imaging was performed on the coronary brain sections, and the spatial distribution of danegapide could be observed by cleaning the obtained ion images. The effect of danegapide on neuronal function could be clearly understood by comparing the control group with the stroke group (Freitas-Andrade, Bechberger et al., 2020). Mass spectrometry imaging plays an important role in assessing the protective mechanisms of drugs. Mirko Muzzi explored the bioenergetics of D-pyrrole in stroke, using mass spectrometry to evaluate the effects of D-pyrrole on bioenergetics, Ca^2+^ flux, electrophysiological function, and death in primary nerve culture and exposure to oxygen-glucose in hippocampal sections. The distribution of D-pyrrole in ischemic brain confirmed that D-pyrrole can bind F1FoATP synthase and increase mitochondrial ATP production, which may play a certain role in experimental ischemic brain injury. Dexamethasone promotes mitochondrial ATP production in cultured neurons or glial cells, reduces energy depletion, prevents intracellular Ca^2+^ overload, and provides cell protection to OGD during culture exposure. The drug can also fight ATP depletion, mitochondrial swelling, hypoxia depolarization, loss of synaptic activity and neuron death in hippocampus brain slices caused by OGD. Ost-ischemia therapy with D-pyrrole reduced the size of cerebral infarcts, and the concentration of D-pyrrole achieved in the ischemic penumbra was equivalent to the neuroprotective concentration found *in vitro* (Muzzi, Gerace et al., 2018). Cilnidipine has been reported to show antihypertensive and a neuroprotective effect in a rat model of cerebral ischemia, but has little distribution in the normal brain, suggesting that it is inhibited by normal brain absorption by efflux transporters such as P-glycoprotein (P-gp). Compared with control LLC PK1 cells, the intracellular accumulation of Cilnidipine was reduced in P-gp overexpressing pig kidney epithelial cells (LLC-GA5-COL150 cells), and this decrease was significantly inhibited by the P-gp inhibitor Verapamil. In addition, p-Gp knockout mice showed a significantly increased concentration of Cilnidipine in the brain after administration of Cilnidipine compared with wild-type mice. The concentration of Cilnidipine in the ischemic hemisphere was 1.6 times higher than that in the lateral hemisphere when it was administered to male spontaneously hypertensive rats (SHR) and obstructed the distal central and ipsilateral common carotid arteries. This result is supported by visualizing the Cilnidipine distribution using MALDI-Tof/MS imaging. The results showed that Cilnidipine is normally excluded from the brain by P-gp-mediated efflux transport, but P-gp function is impaired in the ischemic brain, and therefore Cilnidipinee is distributed in the ischemic region (Yano, Takimoto et al., 2014).

## Mass Spectrometry Imaging Is Used to Reveal the Mechanisms of Traditional Medicines in Cerebral Ischemia

As MALDI-MSI has the characteristics of high throughput *in-situ* imaging, its application in the research of central nervous system diseases and related drug therapy is helpful to explore the mechanism of disease and drug. For clinical treatment, it is urgent to use drugs for chemical intervention in cerebral ischemia-reperfusion injury to promote the recovery of neurological function. Notoginsenoside R1, Dl-3-n-butylphthalide (NBP) and thymoquinone have been found to have therapeutic effects on cerebral ischemia. Ting Zhu’s study explored the potential mechanism of notoginsenoside R1 (NG-R1) on the regulation of small molecule metabolism after ischemic stroke. It was found that NG-R1 reduced infarct size by improving neuronal injury and inhibiting glial activation in MCAO/R rats. Frozen sections of brain tissue were imaged using MALDI-MSI and detailed structural confirmation of the identified metabolites was performed simultaneously. According to the results of mass spectrum imaging, NG-R1 can reduce the abnormal accumulation of glucose and citric acid for 7 days after MCAO/R surgery, and NG-R1 can increase the contents of glutamate and malate aspartic acid, ATP metabolism, antioxidant content and phospholipid content after MCAO/R surgery. NG-R1 regulates atP-metabolism tricarboxylic acid (TCA) cycle malate-aspartate transport antioxidant activity and iron and phospholipid homeostasis in the striatum and hippocampus of MCAO/R rats, and protects the brain from ischemia/reperfusion injury by regulating brain small-molecule metabolism (Zhu, Wang et al., 2020).

Dl-3-n-butylphthalide (NBP) is a drug used in the treatment of ischemic stroke. Run-Zhe Liu systematically studied the effects of Dl-3-n-butylphthalide on small molecule brain metabolism. The spatial distribution of small molecules in the brain of dl-3-N-Butylphthalide treated rats was detected by matrix-assisted laser desorption ionization time of flight mass spectrometry (MALDI-TOF-MS). MALDI-TOF-MS was used to observe the content and distribution of small molecules related to the metabolism of focal cerebral ischemia. These small molecules were found to be involved in glucose metabolism, ATP metabolism, glutamate-glutamine cycle, malate aspartate shuttle, oxidative stress, and inorganic ion homeostasis. Among the thirteen metabolites identified by MALDI-TOF-MSI, seven were further verified as ATP, ADP, AMP, GMP, N-acetyl aspartic acid, ascorbic acid and glutathione. These results showed that Dl-3-n-butylphthalide significantly improved ATP metabolism, antioxidant levels and sodium potassium balance in MCAO rats. Imaging showed that MCAO caused severe damage to the cerebral cortex and the right striatum. The level changes of many small molecules involved in metabolism indicated metabolic abnormalities in severe injured areas. Dl-3-n-butylphthalide mitigated the abnormal changes of these small molecules in the ischemic brain region, thereby improved the metabolism in ischemic brain region (Liu, Fan et al., 2017).

Thymoquinone is one of the main components present in *Nigella glandulifera* Freyn et Sint., known to have multiple biological functions in inflammation, oxidative stress, cancer, aging and lowering blood sugar levels. The nerve protective effect and regulation of small molecule metabolites in cerebral ischemia-reperfusion injury are also found to play a role. The role of Thymoquinone was investigated by exploring the changes of small molecular metabolites in the brain by MALDI-MSI. Thymoquinone was found to significantly improve neurobehavioral scores, reduce infarct size, reduce cerebral edema, and increase the number of normal neurons after injury. MALDI-MSI showed that Thymoquinone reduced abnormal accumulation of glucose, citric acid, succinic acid, and potassium ions. Thymoquinone also increases the number of energy-related molecules (ADP, AMP, GMP, and creatine), antioxidants (glutathione, ascorbic acid, and taurine), and other metabolically related molecules (glutamate, glutamine, aspartic acid, and n-acetyl groups). In conclusion, based on the neuroprotective effect of Thymoquinone on cerebral ischemia-reperfusion injury, the regulation of thymoquinone on energy metabolism and small molecule metabolism was revealed (Tian, Liu et al., 2020).

At present, there are few studies on the application of MSI in the treatment of cerebral ischemia with traditional medicines. The combination of traditional medicines and new technologies is crucial to elucidate the mechanism, which is helpful for us to generate new understanding and discovery on the treatment of cerebral ischemia with traditional medicines. Firstly, current MSI can be divided into molecular imaging of drugs in whole animals and individual tissue imaging. Compared with tissue imaging, the MSI of molecular for drug analysis *in vivo* of whole animals based on Air flow-assisted Ionization (AFAI) is easy to use and doesn’t need to be operated in a high vacuum system. In particular, for drug imaging analysis of whole animals such as rats, the information obtained is more comprehensive and richer (Luo, He et al., 2013). Therefore, we can directly observe the targeted distribution of small molecules by using mass spectrometry, and then accurately analyze the target tissues. It has a guiding function for the next research. Secondly, it is a commonly used method to study the mechanism of drug through cerebral ischemia-reperfusion injury rat model. Conventional pharmacological methods such as TTC staining, HE staining and Nissl-bodies observation were used for detection. The pathological mechanisms involved in cerebral ischemia include oxygen free radical damage and mitochondrial damage, which ultimately lead to neuronal apoptosis. As for the tricarboxylic acid cycle and ATP energy metabolism involved in the mechanism of mitochondrial injury caused by cerebral ischemia, dynamic energy changes of ATP/ADP in ischemic brain can be obtained by mass spectrometry imaging, and neuronal apoptosis can be detected by TUNEL staining and flow cytometry. Ultrastructural changes of brain tissues were detected by transmission electron microscopy, and metabolomics analysis of drug molecular effects was performed by LC-MS. Finally, related proteins and genes were detected by RT-PCR, WB and immunofluorescence. Mitochondrial damage leads to excessive production of reactive oxygen species and active nitrogen, so the related factors mainly include SOD, iNOS, etc. The Bcl-2 protein family is mainly involved in the mitochondria mediated apoptosis pathway. In normal cells, the Bcl-2 protein family and Bax protein family are in equilibrium and do not undergo apoptosis. Apoptosis induced by cerebral ischemia leads to changes in the ratio of Bcl-2 to Bax, so Bax and Bcl-2 are common genes associated with the mechanism of apoptosis. In conclusion, mass spectrometry imaging technology and traditional methods are helpful to further reveal the mechanism of traditional medicine in the treatment of cerebral ischemia.

## Conclusion

In summary, we conclude the different types and principles of mass spectrometry imaging. The application of mass spectrometry to physiological substances such as lipids, neurotransmitters, and the tricarboxylic acid cycle related substances are described. We described the application of mass spectrometry to metabolomics, drug distribution, and the treatment of cerebral ischemia with traditional drugs. MSI has shown great advantages in the study of neurodegenerative diseases, especially cerebral ischemia, and has become a powerful tool in the study of the pathogenesis and drug therapy of cerebral ischemia.

## Discussion

In this paper, we mainly study the application of mass spectrometry imaging to physiological substances, metabolites, drug distribution and traditional drug distribution *in vivo*. In the physiological section, we mainly describe the application of MSI to lipids, neurotransmitters and products related to the energy metabolism of the tricarboxylic acid cycle, current research focuses on the spatial distribution and level dynamics of different types of lipids, neurotransmitters and energy metabolites in brain regions. The research of metabonomics mainly uses mass spectrometry imaging to find metabolic biomarkers, combines metabonomics and proteomics techniques to analyze different metabolic pathways, and further analyzes the unique metabolic pathways. Current studies in the drug distribution section include the use of mass spectrometry imaging to determine the brain distribution of Danegapid, D-Pyrrole and Cilnidipinee. The traditional medicine section describes the involvement of MSI in elucidating the mechanisms of Notoginsenoside R1, DL-3-N-Butylphthalide (NBP) and Thymoquinone in the treatment of cerebral ischemia, The physiological processes involved mainly include energy metabolism and peroxide-related ATP, AMP,GMP, glutathione, etc.

Although mass spectrometry imaging can help consolidate the advantages of omics techniques in the development of biomarkers and accelerate the transition from routine analysis to clinical practice, however, there are still some challenges in using mass spectrometry to detect and popularize markers: 1)Samples generally require complex pre-treatment processes such as enzymatic digestion and isotope labeling, which are time-consuming and require strict standard operating procedures; 2)A variety of instrument platforms and ionization technologies coexist, each with its own advantages and limitations, and it is difficult to form a relatively consistent method such as genome research; 3)Due to the current technical bottleneck, the characteristic peptide analysis cannot fully represent the protein level, and a single mass spectrometry analysis can only analyze one specimen, which limits the clinical detection flux; 4)The large database generated by mass spectrometry requires a more complete data analysis strategy, and the comparability and repeatability of results between different research groups are poor, requiring a large number of samples to verify. While mass spectrometry still faces challenges, we expect these issues to be addressed as the technology matures.

The current status of mass spectrometry imaging detection of physiological substances and metabolites is mainly that the molecular coverage provided by a single MSI platform result in a limited ability to detect and image several classes of molecules in complex samples, such as neutral lipids. Multiple reaction monitoring (MRM) uses unique precursor ion-product ion transitions to add specificity which leads to higher selectivity. And the combination of mass spectrometry and other techniques can improve the accuracy of tissue imaging results. Laser desorption ionization multimodal imaging methods for silicon nanopost arrays (NAPA-LDI or NAPA) already exist, in which single tissue sections are continuously imaging under low and high laser effects, producing the eigenspectra of MALDI and NAPA ionization, respectively, providing supplementary coverage of MALDI by improving the detection of neutral lipids and some small metabolites (Fincher, Korte et al., 2020). MSI combined with a novel analytical platform for liquid chromatography-mass spectrometry (LC-MS), can elucidate a more comprehensive metabolic behavior with temporal and spatial information in tissues. The coronal sections of mouse brain can be imaged by mass spectrometry to compare the spatially metabolites extracted from three different brain regions, including the whole cortex (CTX), hippocampus (HI) and striatum (CPu), were measured by liquid chromatography-mass spectrometry. There is a relatively high correlation between mass spectroscopy and LC-MS data in the whole cortex and striatum. Demonstrating that the combination of the two MS platforms visualizes different temporal and spatial metabolites during the pathological process will help to understand the complex pathogenesis of ischemia-reperfusion (Irie, Fujimura et al., 2014). In IMS studies, emphasis is placed on the use of complementary methods to calibrate metabolite abundance and compare IMS results with biochemical data obtained through different methods to help identify potential artifacts (Dienel 2020). Combined with classical histology, MSI allows drugs, metabolites, lipids, peptides, or proteins to be associated with histopathological features or tissue substructures. IMS has great potential in detecting molecular regulation compared with conventional histochemical, for example, tissues and cells derived from the central nervous system, for understanding the mechanisms of neurodegenerative diseases (Michno, Wehrli et al., 2019). There are many examples of combining MSI with other imaging modalities such as fluorescence, confocal Raman spectroscopy and MRI. For example, brain pathophysiology has been studied using MRI and MSI to establish a correlation between *in vivo* and *in vitro* molecular imaging techniques. Endogenous metabolites and small peptide regulation were assessed according to disease status. MSI is combined with mature molecular imaging techniques to advance the understanding of biological and pathophysiological processes (Porta Siegel, Hamm et al., 2018). MALDI-MSI is increasingly being used to study neurodegenerative and psychiatric diseases at the molecular level, including Alzheimer’s disease (AD), Parkinson’s Disease (PD), and schizophrenia (SCZ) (Schubert, Weiland et al., 2016). The role of proteins in neurodegenerative diseases such as cerebral ischemia could be elucidate by mass spectrometry and peptide spectra. Different proteomic workflows for sample processing used to isolate/enrich specific cell populations or brain regions. (Scifo, Calza et al., 2017). Mass spectrometry imaging has repeatedly been shown to reveal general spatial information about the distribution of proteins and peptides in the pathological brain. MALDI-MS provides insight into the accumulation patterns of different Aβ peptide truncations in plaques in different brain regions and is increasingly suitable for clinical research in the biomedical field (Michno, Wehrli et al., 2019).

The current status of mass spectrometry imaging detection of traditional medicines is mainly that MSI has become more and more widely used in quality control, mechanism and active components of traditional medicines. Molecular imaging of drugs *in vivo* is the key to study the target of drug action. Imaging analysis of multiple types’ compounds can visually and relatively quantitatively reflect the characteristic distribution of molecules in animal tissues by Air Flow-Assisted Ionization IMS Method (AFAI) (Luo, He et al., 2013), which is a new mass spectrometry molecular imaging method. It is more suitable for high quality and high sensitivity molecular imaging analysis of small molecule compounds such as drugs because of on matrix. And it also strengthens the correlation between drug and biomarker distribution *in vivo* and *in situ* information. Comprehensive molecular profile information with spatial distribution characteristics and spatio-temporal dynamic changes can be obtained, and functional metabolites related to the mechanism of drug action can be found. It provides a novel and intuitive analytical method for the study of the mechanism of action or toxicological mechanism of drugs or new drug candidates, and even for the study of new drug candidates with multiple targets or unclear targets (He, Luo et al., 2015). Imaging of tissue characteristic molecules is helpful to clarify their active components and mechanisms of action. For traditional drugs, the active components can be extracted and modified to develop new drugs and promote new and convenient clinical treatment of drugs. Simple mass spectrometry imaging technology has some limitations, so it is expected to provide a new way of thinking when combined with other software and traditional pathological biopsy technology. For example, DESI-MSI is time-consuming, which can be combined with molecular pathological analysis techniques such as HE staining, immunohistochemistry, immunofluorescence and other molecular pathological analysis techniques, successively analysis by mass spectrometry location in the same section. Through compared analysis, molecular typing and molecular staging can be carried out more accurately, which is expected to become a new diagnostic tool of molecular pathology. It promotes the development of precision medicine and facilitates the process of personalized clinical diagnosis and treatment. It is not there yet, the technology is not ready, but the idea opens the door to a whole new world.

## Glossary

ADP: adenosine diphosphate
AgNPs: silver nanoparticles
AMP: adenosine monophosphate
APIR: atmospheric pressure infrared
AP-MALDI: atmospheric pressure matrix assisted laser desorption/ionization
ARA: arachidonic acid
BCCAO: bilateral common carotid artery occlusion
BMP: bis(monoacylglycero)phosphate
Cer: ceramide
CL: contralateral
CNS: central nervous system
Cr: creatine
CTX: cortex
Cx43: conjunctions 43
DA: dopamine
DESI-MSI: desorption Electrospray Ionization Mass Spectrum Image
DHA: docosahexaenoic acid
FT-IR: Fourier transform infrared
GABA: γ -aminobutyric acid
GJ: gap conjunctions
GMP: guanylic acid
GNC: gold-nano-coral
GSH: glutathione, r-glutamyl cysteingl + glycine
HE: hematoxylin-eosin
HIP: hippocampus
HPC: hypoxic preconditioning
5-HT: serotonin
IC: infarct
LA-ICP-MS: laser ablation inductively coupled plasma mass spectrometry
LPC: lysophosphatidylcholine
MALDI-MSI: matrix-Assisted Laser Desorption/Ionization Mass Spectrum Image
MCAO: middle cerebral artery occlusion
MPTP: mitochondrial permeability transition pore
MRI: magnetic resonance imaging
MSI: mass spectrometry imaging
MUFA: monounsaturated fatty acids
NAPE: N-acylphosphatidylethanolamine
NAE: N-acylphosphatidylethanolamine
NBP: Dl-3-n-butylphthalide
NE: norepinephrine
NG-R1: notoginsenoside R1
NIMS: nanostructure-initiator mass spectrometry
NMDA: N-methyl-d-aspartic acid
PC: phosphatidylcholine
P- CR: phosphocreatine
PET: positron emission tomography
P-gp: P-glycoprotein
PLA2: phospholipase A2
PNA: N-phenyl 2-naphthylamine
PVP: polyvinylpyrrolidone
PVT: paraventricular nucleus
SDAPCIL: surface desorption atmospheric pressure chemical ionization
SHR: spontaneously hypertensive rats
SIMS: secondary ion mass spectrometry
SOD: superoxide dismutase
TCA: tricarboxylic acid
TTC: 2, 3, 5-triphenyltetrazolium chloride
